# Contribution of the Broiler Breeders’ Fecal Microbiota to the Establishment of the Eggshell Microbiota

**DOI:** 10.3389/fmicb.2020.00666

**Published:** 2020-04-15

**Authors:** Sandrine Trudeau, Alexandre Thibodeau, Jean-Charles Côté, Marie-Lou Gaucher, Philippe Fravalo

**Affiliations:** ^1^NSERC Industrial Research Chair in Meat Safety (CRSV), Faculté de Médecine Vétérinaire, Université de Montréal, Saint-Hyacinthe, QC, Canada; ^2^CRIPA Swine and Poultry Infectious Diseases Research Center, Faculté de Médecine Vétérinaire, Université de Montréal, Saint-Hyacinthe, QC, Canada; ^3^Pôle Agroalimentaire, Conservatoire National des Arts et Métiers (Cnam), Paris, France

**Keywords:** 16S rRNA, animal health, bacterial transfer, broiler breeders, eggshell microbiota, fecal microbiota, public health

## Abstract

In broiler chicken production, microbial populations on the eggshell surface following oviposition are still poorly characterized, though they may significantly impact both poultry and public health. The aim of this study was to describe the microbiota of both broiler breeder hens’ feces and the surface of their eggs to assess the contribution of the parental fecal microbiota to the eggshell microbiota. A total of twelve breeder flocks in Quebec, Canada, were sampled at two different times, and a total of 940 feces and 16,400 egg surface samples were recovered. Using 16S rRNA gene sequencing, we showed that even if the microbiota of both feces and eggshells were mainly composed of the phyla Firmicutes, Actinobacteria, Proteobacteria, and Bacteroidetes, the bacterial community compositions and structures differed between both types of samples. Our results also showed that both the sampling time and the flock identity significantly influenced the alpha- and the beta-diversities of the studied microbiomes. Using a Venn diagram, we showed that 1790 operational taxonomic units (OTUs) were shared between feces and eggshell samples. Sequences associated with genera of potentially pathogenic and spoilage bacteria, *Acinetobacter*, *Campylobacter*, *Escherichia/Shigella*, *Helicobacter*, *Listeria*, *Proteus*, *Pseudomonas*, *Salmonella*, and *Staphylococcus*, were shared between sample types. Some OTUs highly represented in the fecal microbiota and associated with *Lactobacillus* and *Streptococcus* genera, were absent from eggshells, suggesting a selection during the microbiota transfer and/or the potential role of environmental contamination. To the best of our knowledge, this is the first study using 16S rRNA sequencing to describe the contribution of the transfer from the fecal microbial ecosystem of laying breeder hens to the establishment of the microbiota on the surface of laid eggs, as well as the bacterial communities at both the broiler breeder feces and the eggshell levels.

## Introduction

Chicken meat is an important source of high-quality proteins, vitamins and minerals. It is the leanest and most affordable meat product available worldwide, which together explains its economic worth (Hou et al., 2016). Consequently, the broiler chicken industry has grown considerably over the last decades (Leeson and Summers, 2005). However, chickens are also known as important reservoirs of biological hazards, and they often contribute to the transmission of foodborne pathogens (Kaakoush et al., 2014).

The chicken intestinal tract can be colonized by such pathogenic bacteria but also by numerous microorganisms, including commensal and transient bacteria (Videnska et al., 2013), which together compose the intestinal microbiota. The symbiotic host-microbes relationship within the gastrointestinal tract of birds is primordial for the host growth and health (Apajalahti et al., 2004). The avian microbiota has been better understood with the introduction of culture-independent methods, such as 16S rRNA gene amplicons sequencing (Zhu et al., 2002).

In broiler chicken production, microbiota studies have been conducted mainly at the broiler chicken level rather than at the breeder hen level, and they have mainly focused on describing the microbiota composition of different segments of their gastrointestinal tract (Zhu et al., 2002; Lu et al., 2003; Gong et al., 2007), with only a few that included feces (Kaakoush et al., 2014; Videnska et al., 2014; Pauwels et al., 2015; Hou et al., 2016). It has been reported that the avian microbiota composition is affected by multiple factors including birds’ age (Knarreborg et al., 2002; Videnska et al., 2013). Although the effect of age has been demonstrated for the few days old chick (Knarreborg et al., 2002), and between a pullet and an adult commercial laying hen (Videnska et al., 2013), the picture remains unclear for laying hens (e.g., broiler breeder hens). Studies have demonstrated that some specific foodborne and poultry pathogens found on the eggshell surface could infect the hatchlings and therefore affect the growing broiler health as well as the meat products derived from those chickens (Glávits et al., 1984; Cox et al., 1997; Forgetta et al., 2012; Poulsen et al., 2017). Therefore, describing the identity and the diversity of the bacterial communities present at upstream stages of the broiler chicken production pyramid via molecular approaches could help better manage the risk associated with the transmission of those pathogenic microorganisms.

Immediately after oviposition, the egg temperature, which is around 42°C in the hen’s reproductive tract, brutally drops due to its contact with an external colder environment. This creates a negative pressure inside the freshly laid egg which increases the probability for bacteria present on the eggshell surface to penetrate the shell and to contamine the egg content (Gantois et al., 2009). Several studies have reported that the presence of bacterial contamination on the surface of the eggshell could be attributed to various environmental factors such as the type of birds’ housing system (Jones et al., 2016), the laying rate (Chemaly et al., 2009), the presence of food, water, feces, dust (Im et al., 2015), litter (Quarles et al., 1970), and fluff (Sivaramalingam et al., 2013) and/or cuticle’s state (Sparks and Board, 1985). The presence of moist organic matter facilitates survival or even the growth of pathogenic bacteria on eggshells (Gantois et al., 2009). Broiler breeder hens’ fecal microbial communities are among the first bacteria encountered by the egg during and after the egg laying process but the contribution of these microorganisms to the establishment of the microbiota on the egg surface is still unknown.

Bacterial communities present on the eggshell surface are still poorly characterized although they are among the first bacteria encountered by the broiler chickens after hatching and they may impact both poultry and public health. The majority of data available on eggshell bacterial communities comes from research conducted primarily on eggs intended for human consumption and these data come from culture-based studies. The few 16S rRNA gene sequencing studies conducted on wild birds (Shawkey et al., 2009; Lee et al., 2014; Van Veelen et al., 2018) and laying hens (Neira et al., 2017) that have investigated the eggshell microbiota could not document the inter-flocks’ diversity, the diversity between spaced sampling time point, neither could provide information on the contribution of the first steps of the broiler production, i.e., the broiler breeder hens.

Here, we aimed to evaluate the transfer of the parental fecal microbial ecosystem and its contribution to the establishment of the eggshell’s microbiota. To do so, a 16S rRNA gene sequencing approach was used to describe both the fecal microbiota of broiler breeder hens and the microbiota found on the surface of the eggs layed by these birds at two different sampling time points spaced by a 4-week interval and among different flocks.

## Materials and Methods

### Sample Collection

Feces and eggshells from ten flocks of Cobb 500 broiler breeders and two flocks of Ross broiler breeders were sampled twice (*n* = 24) at a 4-week interval, from October 2016 to June 2017 (Supplementary Table S1). Flocks originated from five chicken farms (free run system) in Quebec, Canada. A flock was defined as a group of chickens raised in the same barn over the same period. Sample collection represented a total of 94 pools of fecal material and 1640 eggshell surfaces.

### Feces Sampling

During each visit and for each breeder flock, ten fresh droppings were collected on the pen floor following a stratified sampling plan, pooled together in a plastic container and homogenized. This was done in quadruplicates. A new pair of nitrile gloves was used for the collection of each fecal pool. Immediately after collection, each pooled sample was used to fill a 2 ml Screw Cap Micro tube (Sarstedt AG & Co. KG, Saint-Leonard, Canada) which was transferred in liquid nitrogen for transportation to the laboratory where it was stored at −80°C until further analyses.

### Eggshell Sampling

During each visit and for each breeder flock, 70 eggs were collected directly from the nests following a stratified sampling plan. Each eggshell was swabbed for 1 min with a sterile wipe (Fisher Scientific, Ottawa, ON, Canada) pre-saturated with saline (0.85% NaCl), after which, each egg was returned to its nest. One sterile wipe was used for every 10 eggshells and a new pair of nitrile gloves was used between each wipe. In addition, during each farm visit, an extra sterile wipe pre-saturated with saline serving as a negative control was taken out of the bag and waved in the air in the farm for 30 s, without coming into contact with any surfaces. Immediately after collection, each wipe was transferred to a 50 ml Screw Cap tube (Sarstedt AG & Co. KG, Saint-Leonard, QC, Canada) and frozen in liquid nitrogen for transportation to the laboratory where they were stored at −80°C until later processing.

Each frozen wipe was aseptically transferred in a 24 oz sterile Whirl-Pak Bag (Nasco, Fort Atkinson, WI, United States) containing 20 ml of phosphate buffered saline (PBS) and blended for 90 s using a Seward Stomacher 400C Lab Blender (Cole-Parmer, Montreal, QC, Canada). Each wipe was squeezed and twisted to extract as much liquid as possible. The recovered volume was transferred into a 50 ml Screw Cap tube (Sarstedt AG & Co. KG) and centrifuged in a Sorvall Legend XTR Centrifuge TX-1000 (Fisher Scientific) for 25 min at 4,500 × *g*. The supernatant was discarded and the DNA was extracted.

### DNA Extraction

The total bacterial DNA was extracted using the DNeasy PowerLyzer PowerSoil DNA Isolation Kit (QIAGEN, Toronto, ON, Canada) according to the manufacturer’s instructions with some modifications. A 250 mg (±10 mg) of feces sample or the entire pellet from an eggshell lab wipe, previously resuspended with a bead-beating solution, was transferred to a PowerBead tube with glass beads. Two heat treatments were performed; a first one at 65°C for 10 min and a second one at 95°C for 10 min. Cells were mechanically lysed twice using a Fastprep-24 5G Sample Preparation Instrument (MP Biomedicals, VWR, Ville Mont-Royal, QC, Canada) set at a speed of 6,5 m/s for 45 s, with a 10 min waiting period on ice between both bead-beating runs. The PowerBead tubes were centrifuged at 10,000 × *g* for 5 min at room temperature (20°C) and the remaining steps of the DNA extraction protocol were conducted according to the manufacturer’s instructions. DNA concentrations were measured using the Qubit 3.0 dsDNA broad-range assay (Fisher Scientific) for feces samples, and the Qubit 3.0 dsDNA high sensitivity assay for eggshell samples, both using a DeNovix QFX Fluorometer (Fisher Scientific). Purified DNA samples were stored at −20°C.

### 16S rRNA Gene Amplicon Libraries and Sequencing

The 16S rRNA gene amplicon libraries were prepared using the universal primer pair 515FP1-CS1F ACACTGACGACATGG TTCTACAGTGCCAGCMGCCGCGGTAA and 806RP1-CS2R TACGGTAGCAGAGACTTGGTCTGGACTACHVGGGTWTC TAAT (Caporaso et al., 2012) which amplifies a 292 bp segment of the V4 region. For each feces and eggshell sample, 12 ng of DNA and 14.5 μl of the DNA (<1 ng/μl), respectively, were amplified in a final reaction volume of 30 μl using Invitrogen Platinum SuperFi DNA Polymerase (Fisher Scientific). The amplification was done with an initial denaturation at 95°C for 15 min, followed by 23 cycles including a denaturation step at 95°C for 30 s, an annealing at 55°C for 30 s, an elongation at 72°C for 180 s, and a final extension at 72°C for 10 min. A negative control, H_2_O, and a positive control, the ZymoBIOMICS Microbial Community DNA Standard (Zymo Research, Irvine, CA, United States) were included. A 5 μl volume of each reaction was run on a 2% agarose gel and visualized following staining to confirm presence of the 292-bp amplicon. Barcoding and DNA sequencing were done on an Illumina Miseq PE250 at The McGill University and Génome Québec Innovation Centre (Montréal, QC, Canada).

### Sequence Data Processing

Reads were cleaned and analyzed using Mothur v.1.39.5 following the Miseq standard operating procedure^1^ (accessed April 2018). First, feces and eggshell sequences were analyzed together. Reads from each sample set were combined using the make.contigs command. Sequences containing polymers or ambiguity were rejected using screen.seqs and identical sequences were merged with the unique.seqs command. The remaining sequences were aligned using the Silva reference files, release 128^2^ and the chimeras were removed. Feces and eggshell sequences were segregated using remove.groups. Sequences originating from the control eggshell sample were removed from the eggshell sequences. The new eggshells dataset and the feces dataset were merged into merge_dataset for the remaining analysis using the merge.count and merge.files commands.

### Taxonomic Classification of Sequences

The sequences were classified at the phylum, class, order, family and genus levels based on homology searches using both Silva version 128 and Ribosomal Database Project (RDP) trainset 16^3^ databases. Only the bacterial and archaeal sequences were kept and clustered into operational taxonomic units (OTUs) at a genetic distance dissimilarity of 3% using the classify.otu command.

### Alpha- and Beta-Diversities

For alpha-diversity analysis, the species diversity within a sample, the number of observed OTUs, Inverse Simpson’s and Shannon even indices were calculated using a subsample with the size of the smallest library with 1,000 iterations; feces and eggshell samples were treated separately. The results were compared between groups using Student’s *t*-test (unpaired and paired) and Kruskal–Wallis test with a significance level of 0.05. These statistical analyses were run on GraphPad Prism 8 (GraphPad Software, LaJolla, CA, United States). For beta-diversity analysis, a measure of similarity between sample pairs, a distance matrix with the similarity values for all pairwise comparisons (*t* = Day 0 and *t* = 4 weeks, flocks 1 to 12, feces and eggshells) was created using the Jaccard index based on shared or distinct species, and the Yue & Clayton index which includes the species proportions of both the shared and distinct species. The different groups were statistically compared using the Analysis of molecular variance (AMOVA) with a significance level of 0.05 and visualized using 2D non-metric multidimensional scaling (NMDS). Biomarkers associated with feces or eggshells were highlighted using the linear discriminant analysis effect size (LEfSe). Finally, OTUs rarely observed in the final merge_dataset (nseqs = 1) were removed using the remove.rare command and a Venn diagram was generated to reveal the OTUs shared among groups. Command lines used in Mothur are available at https://github.com/CRSV. Raw sequences can be found on CNBI SRA database under accession number PRJNA602334.

## Results

### Fecal Microbiota

The number of DNA sequences for the 96 feces samples ranged from 39,680 to 105,243 with an average of 62,661 sequences per sample, and a total of 5,876,676 sequences. The vast majority of sequences, 99.8%, were of bacterial origin, whereas 0.2% were from Archaea.

**FIGURE 1:**
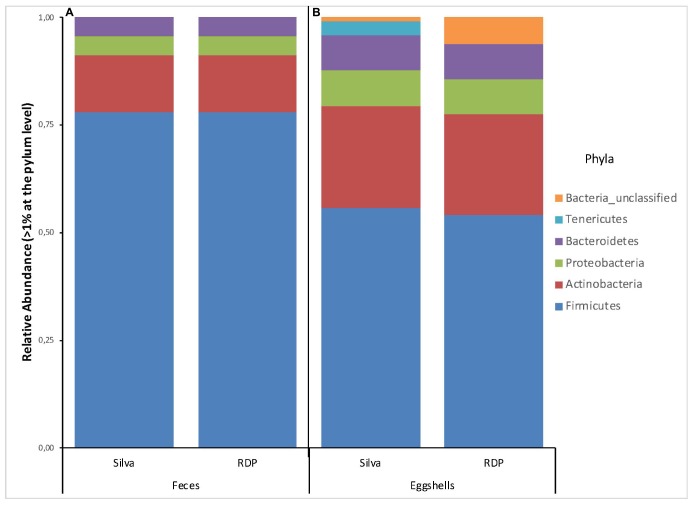
Stacked-bar chart illustrating the major phyla (relative abundance > 1% of total microbiota) found in **(A)** feces, and **(B)** eggshell samples, according to the Silva and RDP databases.

A total of 22 and 20 different phyla were revealed based on searches against the Silva and RDP databases, respectively (Supplementary Table S2). For all feces samples, the number of phyla ranged from 7 to 15 with an average of 12 per sample according to RDP, and from 7 to 18 with an average of 14 per sample according to Silva. Four phyla, Firmicutes, Actinobacteria, Proteobacteria, and Bacteroidetes, showed a relative abundance > 1% of the total fecal microbiota according to each database (Figure 1A). According to both databases, these results are nearly identical.

**FIGURE 2:**
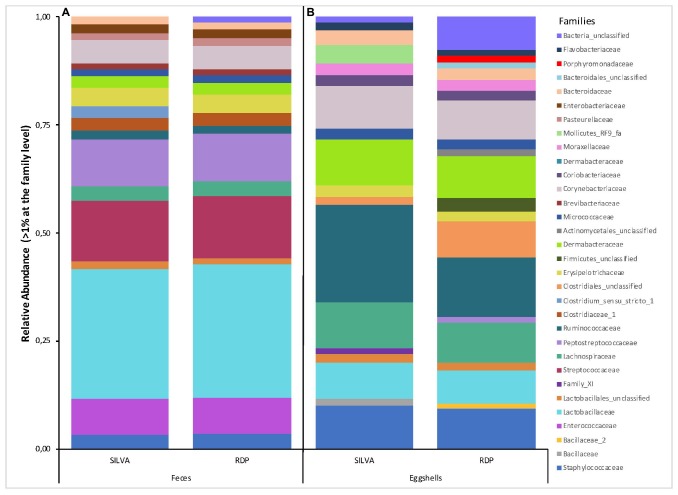
Stacked-bar chart illustrating the major families (relative abundance > 1% of total microbiota) found in **(A)** feces, and **(B)** eggshell samples, according to the Silva and RDP databases.

Likewise, a total of 169 and 193 families were found according to Silva and RDP databases, respectively (Supplementary Table S2). For all feces samples, the number of families ranged from 56 to 91 with an average of 71 according to RDP, and from 60 to 104 with an average of 80 according to Silva. Families with a relative abundance > 1% according to each database are shown in Figure 2A. Again, according to both databases, these results show a high degree of similarity, except for the presence of *Clostridium_sensu_stricto_1* (3%) revealed by Silva and *Bacteria_unclassified* (1%) by RDP.

Genera relative abundance by sample at the genus level was also described at both sampling times (Supplementary Table S4).

### Eggshell Microbiota

The number of DNA sequences from the 168 lab wipes ranged from 546 to 6,216 with an average of 1,233 sequences per lab wipe and a total of 207,225 sequences. Most sequences, 99.6%, were bacteria, and 0.1% were Archaea.

A total of 26 and 29 different phyla were identified based on the Silva and RDP databases, respectively (Supplementary Table S2). For all samples, the number of phyla ranged from 6 to 20 with an average of 11 per sample according to RDP, and from 8 to 22 with an average of 14 per sample according to Silva. The phyla with a relative abundance > 1% of the total eggshell microbiota according to each database are shown in Figure 1B. 6 and 5 phyla were revealed, based on the Silva and RDP databases, respectively. Of these, four phyla, Firmicutes, Actinobacteria, Proteobacteria and Bacteroidetes, showed similar relative abundances according to both databases. The main differences were observed for the relative abundance of Tenericutes (3%) and Bacteria_unclassified (1%) according to Silva and an absence of Tenericutes and a relative abundance of Bacteria_unclassified (6%) when using RDP.

A total of 344 and 257 families were found according to the Silva and RDP databases, respectively (Supplementary Table S2). For all samples, the number of families ranged from 48 to 118 with an average of 72 families according to RDP, and from 49 to 104 with an average of 82 families according to Silva. Families with a relative abundance > 1% according to each database are shown in Figure 2B. Several differences were revealed according to each database, not only in the relative abundance of some families but also in the presence or absence of specific families, most notably the presence of *Mollicutes_RF9_fa*, *Enterococcaceae*, and *Bacillaceae* according to Silva and the presence of *Porphyromonadaceae*, *Bacteroidales_unclassified*, *Actinomycetales_unclassified*, *Firmicutes_unclassified*, *Peptostreptococcaceae*, and *Bacillaceae_2* according to RDP. These differences were mostly attributed to the capacity to assign an identity to the analyzed sequences.

Both databases appear complementary, but because some sequences could not be assigned an identity using Silva and remained unknown, the RDP database was retained for the remaining analyses.

### Detection of Potential Pathogens and Spoilage Bacteria

Potential pathogens and spoilage bacteria of interest in chickens were found in feces and on eggshells. A flock was considered positive when at least one feces or eggshell sample contained a related bacterial sequence. Percentages of positive flocks, positive samples and relative abundance are listed in Table 1. Our results show that in some cases, the percentages of positive flocks, positive samples and relative abundance varied according to the type of samples, feces or eggshell, and the bacterial genus. In most cases, the relative abundances of these genera were very low, ranging from 9.9 × 10^–1^ for *Staphylococcus* to 3.4 × 10^–5^ for *Salmonella* in feces samples and from 1.7 × 10^0^ for *Acinetobacter* to 9.7 × 10^–4^ for *Listeria* on eggshells. Interestingly, members of some bacterial genera, namely *Acinetobacter*, *Campylobacter*, *Proteus*, and *Salmonella*, showed a relative abundance 100-fold higher on eggshells that in feces, and members of *Listeria* and *Pseudomonas* showed a relative abundance 10-fold higher on eggshells than in feces.

**TABLE 1:** Distribution of potential pathogens and spoilage bacterial genera found in the feces and on the eggshells according to the RDP database.

Bacterial genera	Type^1^	Positive flocks (%)		Positive samples (%)		Relative abundance (%)	
		Feces	Eggshells	Feces	Eggshells	Feces	Eggshells
*Acinetobacter*	S	92	100	43	87	2.5 × 10^–2^	1.7 × 10^0^
*Campylobacter*	P	25	75	5	8	2.4 × 10^–4^	1.2 × 10^–2^
*Escherichia/Shigella*	P	100	100	88	22	2.9 × 10^–2^	4.2 × 10^–2^
*Helicobacter*	P	100	83	66	15	1.7 × 10^–2^	2.2 × 10^–2^
*Listeria*	P	33	17	4	1	2.2 × 10^–4^	9.7 × 10^–4^
*Proteus*	P, S	58	83	12	10	3.1 × 10^–4^	1.3 × 10^–2^
*Pseudomonas*	S	100	100	40	75	2.0 × 10^–2^	3.1 × 10^–1^
*Salmonella*	P	17	25	2	3	3.4 × 10^–5^	6.3 × 10^–3^
*Staphylococcus*	P	100	100	100	100	9.9 × 10^–1^	8.6 × 10^–1^

*^1^P, potential bacterial pathogens; S, spoilage bacteria.*

### Alpha- and Beta-Diversities

A total of 25,038 OTUs were found in all 96 feces and 168 eggshell samples. The number of OTUs for the 96 feces samples ranged from 506 to 1,608 with an average of 975 OTUs per sample. Likewise, the number of OTUs for the 168 eggshell samples ranged from 190 to 667, with an average of 358 OTUs per sample.

Comparisons of alpha-diversity indices, Sobs, Inverse Simpson’s and Shannon even, between both visits, at time = Day 0 and after 4 weeks, for each of the 12 flocks, and for both types of samples, feces and eggshells, are listed in Table 2. The flock and sampling time point were shown to have significant effects on alpha-diversity indices.

**TABLE 2:** Comparison of alpha-diversity indices of feces and eggshells across broiler breeder flocks according to the time and the flock.

Broiler breeder groups	Feces			Eggshells		
	Sobs	Inverse Simpson’s	Shannon even	Sobs	Inverse Simpson’s	Shannon even
Flock 1 Day 0 vs. 4 weeks	748	11	0.50	207	51	0.86
Flock 2 Day 0 vs. 4 weeks	**799**	12	0.51	173	13	0.71
Flock 3 Day 0 vs. 4 weeks	893	15	0.50	235	**101**	**0.91**
Flock 4 Day 0 vs. 4 weeks	908	16	0.53	219	62	0.88
Flock 5 Day 0 vs. 4 weeks	**900**	17	0.52	**245**	65	**0.85**
Flock 6 Day 0 vs. 4 weeks	880	18	0.53	**223**	**63**	**0.84**
Flock 7 Day 0 vs. 4 weeks	825	16	0.51	240	85	0.90
Flock 8 Day 0 vs. 4 weeks	740	11	0.48	246	105	0.92
Flock 9 Day 0 vs. 4 weeks	658	12	0.47	253	89	0.91
Flock 10 Day 0 vs. 4 weeks	794	13	0.50	276	97	0.91
Flock 11 Day 0 vs. 4 weeks	719	11	0.47	207	36	0.77
Flock 12 Day 0 vs. 4 weeks	786	14	0.50	251	80	0.88
All Flocks Day 0 vs. 4 weeks*	806*	14*	0.50*	230*	70*	0.86*
All Flocks^1^**	0.09**	0.2**	0.5**	<0.0001**	<0.0001**	<0.0001**

The means were based on 1,000 subsampling of 39,680 and 546 sequences for the feces and the eggshells, respectively. p-value under significant level (p < 0.05) are represented in bold according to an unpaired t-test or a * paired t-test. ^1^Regardless of time. **p-value of a Kruskal–Wallis test.

**FIGURE 3:**
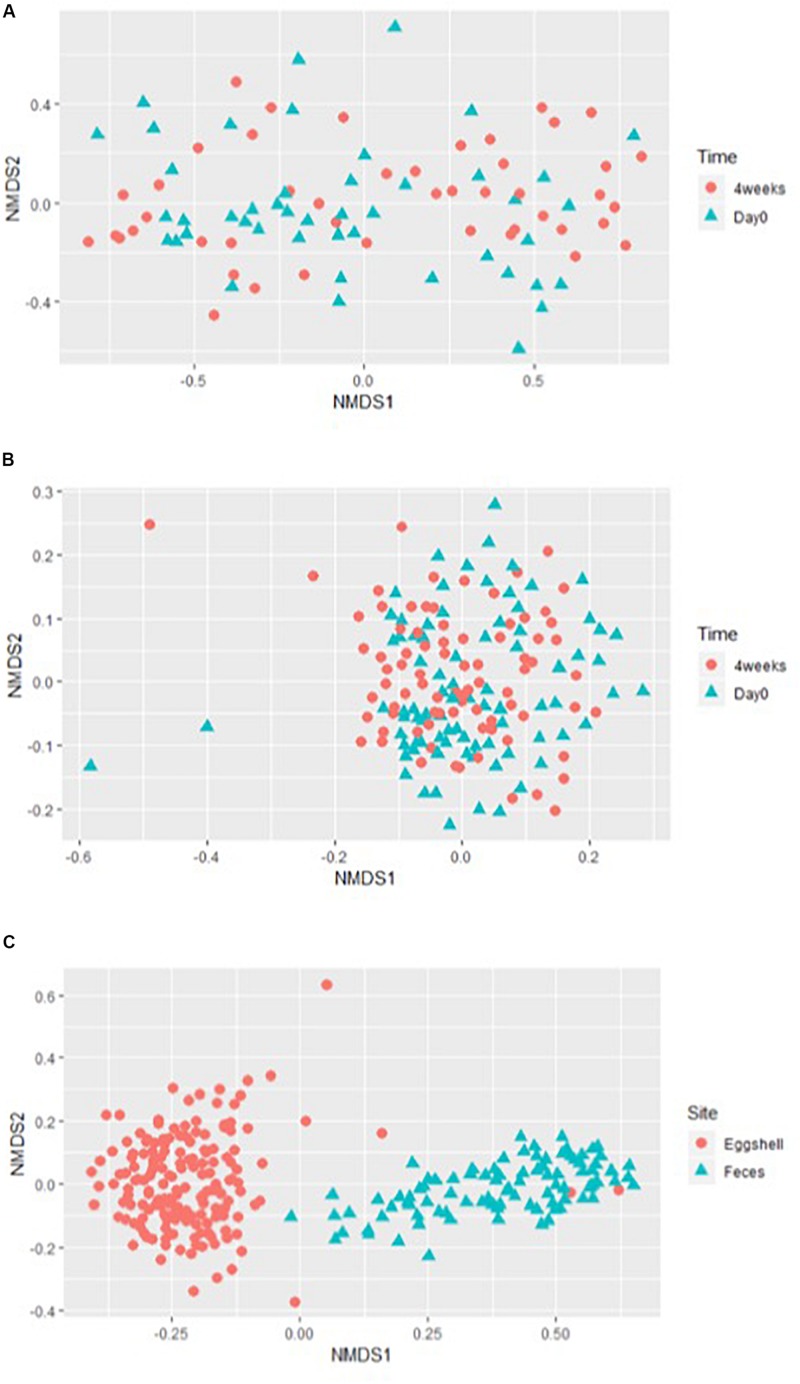
Examples of the 2-dimensions NMDS plots using Jaccard distances matrices to compare **(A)** broiler breeder fecal microbiota beta-diversity according to the time of sampling, Day 0 and 4 weeks; **(B)** eggshell microbiota beta-diversity according to the time of sampling, Day 0 and 4 weeks; and **(C)** overall microbiota according to the sample type, eggshell, and feces.

**FIGURE 4:**
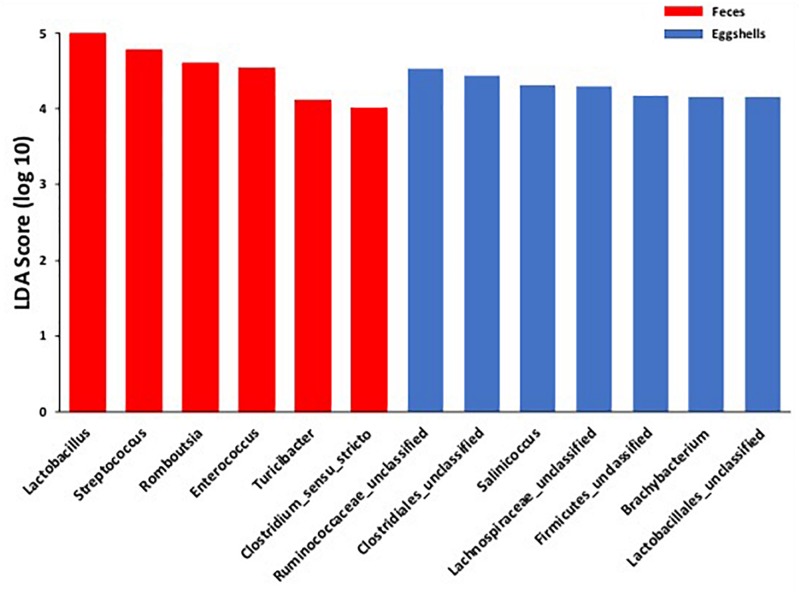
Comparison of the broiler breeder fecal microbiota and eggshell microbiota using LEfSe. Histogram of the LDA scores for significantly differentially abundant biomarkers among groups (LDA Score [log 10] > 4). Red = feces and Blue = eggshells.

For the beta-diversity, the Jaccard and Yue & Clayton indices between both visit time points, Day 0 and after 4 weeks, for each of the 12 sampled flocks, and for both types of samples, feces and eggshells, are listed in Table 3. Our results showed that the sampling time point and the flock had a significant effect on beta-diversity indices. Some of the Jaccard distance matrices were plotted using 2D NMDS in which feces samples at Day 0 and after 4 weeks (Figure 3A), eggshell samples at Day 0 and after 4 weeks (Figure 3B) and feces and eggshell samples (Figure 3C) were compared. All the groups (Day 0 vs. 4 weeks, flock vs. flock, and feces vs. eggshell samples) were statistically compared (Table 3 and Supplementary Table S3). Our results showed that the fecal microbiota and the eggshell bacterial communities significantly evolved during the 4-week interval (*p* < 0.05), and were flock-specific (*p* < 0.001). Moreover, feces and eggshell microbiota were significantly different (*p* < 0.001). LEfSe was used to highlight biomarkers that were the most strongly associated with each type of sample (LDA Score [log 10] > 4). Six genera with high LDA scores indicative of marked abundances in feces, *Lactobacillus*, *Streptococcus*, *Romboutsia*, *Enterococcus*, *Turicibacter*, and *Clostridium_sensu_stricto*, were revealed (Figure 4). Likewise, seven genera with high LDA scores indicative of marked abundances on eggshells, *Ruminococcaceae_ unclassified*, *Clostridiales_unclassified*, *Salinococcus*, *Lachnospiraceae_ unclassified*, *Firmicutes_unclassified*, *Brachybacterium*, and *Lactobacillales_unclassified* were revealed (Figure 4).

**TABLE 3:** Microbiota structure comparison of feces and eggshells across broiler breeder flocks according to the time and the flock.

Compared groups	AMOVA (*p*-value)			
	Feces		Eggshells	
	Jaccard	Yue & Clayton	Jaccard	Yue & Clayton
Flock 1 Day 0 vs. 4 weeks	0.066	0.786	**0.001**	**0.005**
Flock 2 Day 0 vs. 4 weeks	0.035	0.074	**<0.001**	0.484
Flock 3 Day 0 vs. 4 weeks	**0.038**	**0.005**	**0.004**	0.14
Flock 4 Day 0 vs. 4 weeks	**0.047**	**0.006**	**0.001**	**<0.001**
Flock 5 Day 0 vs. 4 weeks	0.287	0.609	**0.004**	**0.002**
Flock 6 Day 0 vs. 4 weeks	**0.044**	0.229	**0.002**	**<0.001**
Flock 7 Day 0 vs. 4 weeks	**0.028**	**0.032**	**0.001**	**<0.001**
Flock 8 Day 0 vs. 4 weeks	**0.006**	**0.039**	**<0.001**	**0.001**
Flock 9 Day 0 vs. 4 weeks	0.096	**0.039**	**0.001**	**<0.001**
Flock 10 Day 0 vs. 4 weeks	**0.024**	**0.032**	**<0.001**	**<0.001**
Flock 11 Day 0 vs. 4 weeks	0.068	**0.032**	**0.001**	**0.017**
Flock 12 Day 0 vs. 4 weeks	0.382	0.368	**0.001**	**0.014**
All Flocks Day 0 vs. 4 weeks	**0.019**	0.86	**0.006**	**0.007**
All Flocks^1^*	**<0.001**	**<0.001**	**<0.001**	**<0.001**
	**Jaccard**		**Yue & Clayton**	
All flocks feces vs. eggshells	**<0.001**		**<0.001**	

p-value under significant level (p < 0.05) are represented in bold. ^1^Regardless of time. *The comparisons between each flock are represented in Supplementary Table S2.

### Transfer of Bacterial Communities

**FIGURE 5:**
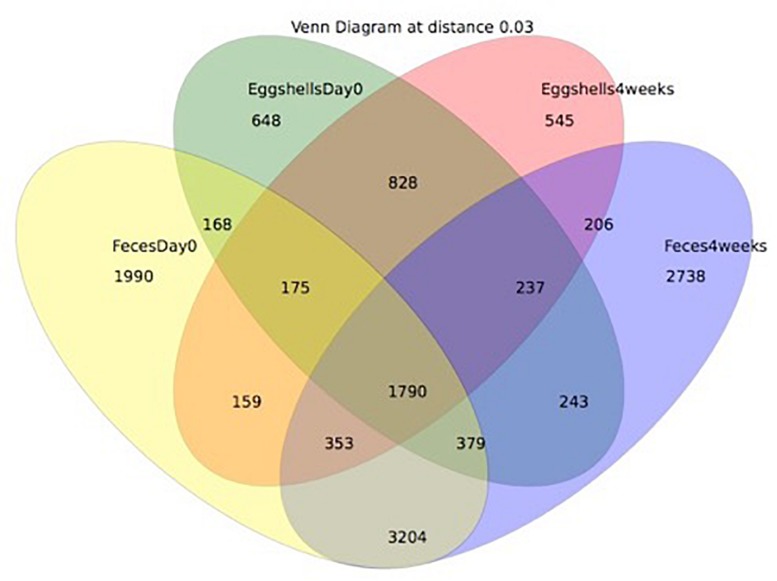
Venn diagram showing the shared OTUs by groups. The groups compared were: (i) eggshells at Day 0 of the study; (ii) eggshells 4 weeks after; (iii) feces at Day 0 of the study; (iv) and feces 4 weeks after.

A Venn diagram was created to show the number of OTUs shared or unique to feces and eggshells for both sampling time points, Day 0 and 4 weeks (Figure 5). A total of 1790 OTUs were common between both sample types, feces and eggshells, and present during both sampling time points (Supplementary Table S5). Of these, some OTUs were found more than 1000 times and belonged to the genera *Lactobacillus*, *Brachybacterium*, *Staphylococcus*, *Lachnospiraceae_unclassified*, *Jeotgalicoccus*, *Bacteroides*, *Ruminococcaceae_unclassified*, *Corynebacterium*, *Salinicoccus*, and *Clostridiales_unclassified*. Likewise, some other OTUs were found more than 500 times, and belonged to the genera *Enterococcus*, *Yaniella* and *Brevibacterium*, *Romboutsia*, and *Bacteria_unclassified*.

Sequences associated with each of the nine potentially pathogenic and spoilage bacterial genera listed in Table 1 were observed in both feces and eggshell samples from at least one of the two sampling time points (data not shown). A flock was considered positive for the presence of potentially pathogenic or spoilage bacteria when at least a sequence related to those bacteria was identified in one of the four feces or in one of the seven eggshell samples, respectively. It is worth noting that in some flocks, some sequences associated with potentially pathogenic and spoilage bacteria, namely *Acinetobacter*, *Listeria*, *Salmonella*, *Proteus*, and *Campylobacter*, were identified on eggshells but could not be found in the feces samples (Table 4). For example, for 58% of the sampled flocks, some sequences associated with the genus *Campylobacter* were identified on eggshells whereas these sequences were not identified from their related feces samples. However, 17% of the remaining flocks harbored some other sequences associated with *Campylobacter* in both feces and eggshell samples.

**TABLE 4:** Percentage of flocks for which genus sequence ^1^ were detected * on both eggshells and feces samples, and for which genus sequence ^1^ were detected * on the eggshells but that were absent in feces.

		Flock (%)	
Genus sequence	Type^2^	Eggshells+/Feces+	Eggshells+/Feces−
*Acinetobacter*	S	92	8
*Campylobacter*	P	17	58
*Escherichia/Shigella*	P	100	0
*Helicobacter*	P	83	0
*Listeria*	P	8	8
*Proteus*	P, S	50	33
*Pseudomonas*	S	100	0
*Salmonella*	P	8	17
*Staphylococcus*	P	100	0

^1^The taxonomic assignment is according to the RDP database. *At least one sample within the flock contained sequences of the bacterial genus. ^2^P, potential bacterial pathogens; S, spoilage bacteria.

## Discussion

Our study describes for the first time the contribution of the chicken’s fecal microbiota to the establishment of the microbiota found on eggshells in a commercial farm. A first step toward documenting the transfer of the parental microbiota to the eggshell was to describe the bacterial community structure of both feces and eggshells, information that was absent in the scientific literature. In addition, our analyses of the broiler breeder fecal and eggshell microbiota were done at two sampling time points and between different flocks.

The broiler breeder fecal microbiota was mainly (>90%) composed of members of the phyla Firmicutes, Actinobacteria, Proteobacteria, and Bacteroidetes. Similar results have been reported for wild birds (Banks et al., 2009; Lu et al., 2009), laying hens (Videnska et al., 2013, 2014) and broiler chickens (Kaakoush et al., 2014; Videnska et al., 2014; Pauwels et al., 2015; Hou et al., 2016). In addition to these 4 major phyla, 19 other phyla were identified from the collected feces samples in our study, including some phyla never previously described, namely Elusimicrobia, Nitrospirae, Planctomycetes, Saccharibacteria, and Hydrogenedentes.

Our results showed that *Lactobacillaceae* was the most dominant family found in feces, which is in agreement with the works of others who described the intestinal microbiota of broiler chickens (Forgetta et al., 2012; Kaakoush et al., 2014; Pauwels et al., 2015; Stanley et al., 2015; Oakley and Kogut, 2016; Yan et al., 2017). The role of *Lactobacillaceae* in the improvement of avian gut health through a modulation of the immune system (De Maesschalck et al., 2015) suggests that birds with higher relative abundance of this family, within a diverse microbiota, might be healthier than those with lower proportions of *Lactobacillaceae* (Singh et al., 2012; Deusch et al., 2015). *Streptococcaceae* and *Peptostreptococcaceae* were also the most abundant families in the Firmicutes, which is in agreement with Kaakoush et al. (2014). However, *Ruminococcaceae* represented only 1% of the families identified in the present work which is in contradiction with the results of others who showed this family to be dominant in broiler chicken feces (Singh et al., 2012; Kaakoush et al., 2014; Videnska et al., 2014). Some studies indicated that the presence of *Ruminococcaceae* was negatively correlated with the feed conversion ratio of the birds and therefore with the weight gain (Singh et al., 2012; Menni et al., 2017). Broiler breeders are genetically selected for their high conversion index which supports the fact that the birds sampled in our study had low proportions of *Ruminococcaceae* in their fecal microbiota. Our results also revealed that *Enterobacteriaceae*, *Corynebacteriaceae*, and *Bacteroidaceae* dominated, respectively, the Proteobacteria, Actinobacteria and Bacteroidetes phyla, an observation also made by others (Singh et al., 2012; Kaakoush et al., 2014; Videnska et al., 2014). In addition to corroborating the information of the few studies on birds’ fecal microbiota, our results also revealed greater bacterial community richness.

Most published information on eggshell microbiota come from culture-based studies. It has been reported that a clean eggshell harbored 10^3^ bacteria per egg (Sauveur, 1978). Only a few studies have investigated the eggshell microbiota using culture independent high-throughput sequencing methodology (Shawkey et al., 2009; Lee et al., 2014; Neira et al., 2017; Van Veelen et al., 2018). Our work identified a total of 37 phyla from the chicken eggshell surface. A similar richness was reported for eggs laid by wild birds (Shawkey et al., 2009; Lee et al., 2014), and a lower richness, 22 phyla, was reported for eggs laid by commercial laying hens living in a free-range environment (Neira et al., 2017). Our work also indicated that more than 90% of the eggshell microbiota was represented by members of the Firmicutes, Actinobacteria, Proteobacteria, and Bacteroidetes phyla, also all identified as dominant phyla in the feces samples analyzed. Similar microbiota composition was also reported on the eggshell of wild birds (Lee et al., 2014) and of laying hens from free-range system (Neira et al., 2017). Interestingly, for cage-housed hens (Neira et al., 2017), Fusobacteria was reported as the second predominant phylum, suggesting a role of the hen housing system on eggshell bacterial major communities. With the exception of two phyla, namely Nitrospinae and Thermodesulfobacteria, the phyla reported by Neira et al. (2017) were also identified in the present study. In addition, our work revealed 10 phyla that had never been associated with eggshells, namely Candidatus Saccharibacteria, Euryarchaeota, Parcubacteria, SR1, Thaumarchaeota, FBP, RsaHf231, TM6_(Dependentiae), BJ-169, and Candidate_division_WPS-1.

Our results showed that at least 257 families were found on the eggshells sampled, a richness similar to the one reported for wild birds’ eggshells (Shawkey et al., 2009). However, the identity of the major families clearly differed between our study and theirs. Our work showed that *Ruminococcaceae* was the most abundant member of Firmicutes, while *Lachnospiraceae* was the dominant family in the work from Neira et al. (2017) on free-range birds. To the best of our knowledge, some of the major families identified in our study have never been described on eggshells, i.e., *Bacillaceae_2*, *Family_XI* and *Mollicutes_RF9_fa*. Factors such as the breed, the production system and the sampling procedure could all contribute to these differences (Neira et al., 2017).

In our study, potential animal and human pathogens (Lu et al., 2003; Hameed and Amin, 2010; Crespo et al., 2013; Nasrin et al., 2013; Thomas et al., 2013; Ebringer and Rashid, 2014; Humphrey et al., 2014; Rouger et al., 2017), and spoilage bacteria (Techer et al., 2013; Neira et al., 2017), identified at the genus level, were reported from both the feces and eggshell samples analyzed. Even if most genera were present in low abundances, they can have important consequences on animal health, and ultimately on public health. For feces, studies conducted on broiler chickens also identified some of these genera in fecal (Chien et al., 2011; Singh et al., 2012; Yan et al., 2017) or cecal samples (Zhu et al., 2002) with different relative abundances. Eggshells from laying hens presented these genera in different relative abundances (Neira et al., 2017) or detection rates (Cook et al., 2003; Jones et al., 2012). Interestingly, according to our results, some genera were over represented in eggshell microbial communities when compared to fecal ones. Considering that the fecal bacterial communities contribute to the contamination of the eggshell, this overrepresentation suggests a non-homogeneous transfer, perhaps indicative of a bacterial selection. The factors contributing to the selective transfer and survival of specific bacterial taxa on eggshells warrants further investigation, potentially through cultivation-dependent methods to confirm survivorship and provide a means to investigate traits which facilitate survival.

The alpha-diversity of feces and eggshell microbiotas were differentially affected by the sampling time and by the flock. For feces, alpha-diversity changes were supported only by the sobs index and only for some specific flocks. Significant changes in richness and community diversity among the two sampling time points were previously reported in a study conducted on broiler chickens (Oakley and Kogut, 2016). These differences suggest that the alpha-diversity of the fecal microbiota is more stable in adult laying hens than in younger chickens. For eggshells, alpha-diversity appeared strongly affected by the flock and to a lesser extent by the sampling time, an observation that had not been previously reported. Differences were observed between flocks housed on the same farm (Supplementary Table S3), highlighting the fact that the flock as a variable must be taken into account when designing microbiota experiments that will be used to answer a precise research question.

We also showed that the beta-diversity of feces and eggshell microbiota was affected by both the sampling time and the flock. For feces, some studies reported significant differences over time for broiler chickens’ fecal microbiota (Van Der Wielen et al., 2002; Sekelja et al., 2012; Oakley and Kogut, 2016), but this parameter had not been investigated for adult breeder hens. Some authors reported inter-flock fluctuations for commercial laying hens (Videnska et al., 2013) and broiler chickens (Kaakoush et al., 2014; Hou et al., 2016). These fluctuations were associated to different parameters such as the origin of the birds (Videnska et al., 2014), and the type of litter or feed (Stanley et al., 2015). In our study, differences over time were supported by the presence of minor communities according to the Jaccard index which considers the presence or absence of specific OTUs, which is highly relevant when considering pathogenic bacteria. For eggshells, our results showed, for the first time, an evolution of the egg surface microbiota structure within a specific flock over a 4-week interval. From a commercial perspective, our results suggested that the hatching chickens could be exposed to different bacterial populations, depending on the flock of origin, and the moment during the lay period.

Moreover, according to the LEfSe, OTUs classified as *Lactobacillus* and *Streptococcus* genera, were identified as biomarkers of the fecal microbiota. These results reinforce the hypothesis of a selection during the microbiota transfer, perhaps owing to the differential capability of some bacteria to attach to or survive on the eggshell surface.

These analyses could also suggest that some OTUs identified on eggshells did not originate from feces. Most OTUs systematically shared between feces and eggshells were previously reported from the gastrointestinal tract and/or in the feces of broiler chickens (Lu et al., 2003; Hou et al., 2016; Le Gall-David et al., 2017; Qiao et al., 2018). Others were also identified in samples from the poultry farms environment, notably in air, litter, and dust samples (Collins et al., 1988; Weidhaas et al., 2010; Martin et al., 2011; O’Brien et al., 2016). A recent study involving passerine birds revealed that the eggshells had richer and more diverse bacterial communities than those found in the female cloaca. These authors identified the skin and feather bacterial communities as major contributors to the eggshell microbiota (Van Veelen et al., 2018). Another study conducted on Eurasian Magpie reported that the eggshell microbiota was largely influenced by the microorganisms found in the nests (especially the feathers), whereas the maternal gut or cloaca appeared to be minor contributors (Lee et al., 2014). In our study on broiler breeders, the 1790 shared OTUs represented 31% of the OTUs detected in eggshell samples and 15% of the OTUs found in feces samples, suggesting again that sources other than feces could contribute to the microbiota found on the eggshell in commercial breeder hens.

For numerous OTUs found on eggshells, including potentially pathogenic bacteria, e.g., *Listeria*, *Salmonella*, *Proteus*, and *Campylobacter*, their presence on the egg surface could not be explained by their presence in feces, which suggests again an environmental contribution. A study on wild birds suggested that parental care could affect the eggshell microbiota by protecting the egg through an increase in the abundance of some antibiotic-producing bacteria, e.g., *Bacillus*, on the eggshells, or by transferring these bacteria from their feathers (Lee et al., 2014). As the poultry production and its environment appear to be a favorable reservoir for *Proteus* (Yeh et al., 2018), *Listeria* (Dahshan et al., 2016), *Salmonella* and *Campylobacter* (Jones et al., 2016), improved biosecurity measures would contribute in preventing the colonization of the hatchlings by pathogens which contaminate the eggshell.

The current study identified several potential pathogens and spoilage bacteria that were shared between the broiler breeder feces and the surface of their laid eggs such as *Acinetobacter*, *Campylobacter*, *Escherichia*, *Helicobacter*, *Listeria*, *Pseudomonas*, *Proteus*, *Salmonella*, and *Staphylococcus*. For *Campylobacter*, a recent research work conducted on laying hens also reported this sharing for birds living in cage-free systems (Jones et al., 2016). A recent study reported the transmission of *Escherichia* from the breeder parents (fecal samples) to the shell of their laid eggs (Projahn et al., 2016). For *Salmonella*, it is known that the bacterium can contaminate the eggshell, and for some serotype, the inside of eggs through an infection of the hen’s reproductive tract or feces (Gantois et al., 2009). For *Pseudomonas*, it has been reported that the watery content of the fecal material which contaminates the egg surface would increase the capacity of the bacterium to digest the cuticle, giving bacteria better access to the pores, and consequently, increasing the risks of internal contamination of the egg by pathogens (Cook et al., 2003, 2005). Given the possible involvement of these bacteria in diseases or in food spoilage, these sharing are relevant both in terms of bird health, egg conservation and food safety.

## Conclusion

Our study documented for the first time the contribution of the fecal microbiota of commercial broiler breeders to the establishment of the microbiota found on the surface of their laid eggs. This work also provided the first description of feces and eggshell microbiota of broiler breeders using high throughput sequencing methodology. Our results showed that these microbiomes evolved over time and varied among flocks under these conditions. Our results also revealed that the presence of potentially pathogenic bacteria on the eggshell was not always related to their presence in fecal matters. Therefore, it is important that all stakeholders, including producers, breeders, veterinarians, inspectors and researchers, be aware of the microorganisms present upstream in the broiler breeder production chain in order to better manage the risks for poultry and public health.

## Data Availability Statement

The datasets generated for this study can be found in the Raw sequences can be found on CNBI SRA database under accession number PRJNA602334.

## Ethics Statement

The experimentations presented in this manuscript did not require the approval of an ethic committee as they did not involve the manipulation of birds. Strict biosecurity measures were followed at each visit and all sampling were in accordance with and under the supervision of a representative of the establishments visited. Freshly voided feces were collected without the manipulations of birds and eggs were gently sampled.

## Author Contributions

AT, M-LG, and PF designed the experiments. ST performed the experiments. ST, AT, and PF analyzed the results. ST, AT, M-LG, J-CC, and PF discussed the results and wrote the manuscript.

## Conflict of Interest

The authors declare that the research was conducted in the absence of any commercial or financial relationships that could be construed as a potential conflict of interest.

## Footnotes

1 https://www.mothur.org/wiki/MiSeq_SOP

2 https://www.mothur.org/wiki/Silva_reference_files

3 https://www.mothur.org/wiki/RDP_reference_files

## Supplementary Material

TABLE S1 Information on the broiler breeder flocks.

TABLE S2 Total phyla, families and genera found in feces and/or eggshell samples according to Silva and RDP databases.

TABLE S3 Microbiota structure comparison of **(A)** feces and **(B)** eggshells across broiler breeder flocks according to the flock using the Jaccard and Yue & Clayton indices.

TABLE S4 Genera relative abundance by sample at the genus level at both samplng times.

TABLE S5 Taxonomy identification and number of sequences associated with OTUs systematycally shared by feces and eggshell samples according to Silva and RDP databases.
