# Supplementary material for: Contribution of the Broiler Breeders’ Fecal Microbiota to the Establishment of the Eggshell Microbiota
Source: Front Microbiol. 2020 Apr 15;11:666. doi: 10.3389/fmicb.2020.00666 (PMC7176364; doi:10.3389/fmicb.2020.00666)
Supplement: TABLE S1 — Information on the broiler breeder flocks. [file Table_1.PDF]

Supplementary Table S1. Information on the broiler breeder flocks.

Flock ID	Farm ID	Chicken breed	Age at sampling time Day 0	Age at sampling time 4 weeks	Number of birds / flock
Flock 1	Farm A	Ross	36-week-old	40-week-old	NA
Flock 2			45-week-old	49-week-old	NA
Flock 3	Farm B	Cobb 500	40-week-old	44-week-old	14,134
Flock 4	Farm C				13,933
Flock 9	Farm D				19,800
Flock 10					18,629
Flock 5	Farm B		28-week-old	32-week-old	11,916
Flock 7	Farm C				11,673
Flock 8					6,893
Flock 6	Farm B				5,575
Flock 11	Farm E				8,098
Flock 12					11,200
