# Supplementary material for: Contribution of the Broiler Breeders’ Fecal Microbiota to the Establishment of the Eggshell Microbiota
Source: Front Microbiol. 2020 Apr 15;11:666. doi: 10.3389/fmicb.2020.00666 (PMC7176364; doi:10.3389/fmicb.2020.00666)
Supplement: TABLE S3 — Microbiota structure comparison of (A) feces and (B) eggshells across broiler breeder flocks according to the flock using the Jaccard and Yue & Clayton indices. [file Table_3.PDF]

Supplementary Table S3. Microbiota structure comparison of A) feces and B) eggshells across broiler breeder flocks according to the flock using the Jaccard and Yue & Clayton indices.

A)	Flock 1 / Farm A	Flock 2 / Farm A	Flock 3 / Farm B	Flock 4 / Farm C	Flock 5 / Farm B	Flock 6 / Farm B	Flock 7 / Farm C	Flock 8 / Farm C	Flock 9 / Farm D	Flock 10 / Farm D	Flock 11 / Farm E	Flock 12 / Farm E
Flock 1 / Farm A		**<0.001**	**<0.001**	**<0.001**	**<0.001**	**<0.001**	**<0.001**	**<0.001**	**<0.001**	**<0.001**	**<0.001**	**<0.001**
Flock 2 / Farm A	0.305		**<0.001**	**<0.001**	**<0.001**	**<0.001**	**<0.001**	**<0.001**	**<0.001**	**<0.001**	**0.001**	**<0.001**
Flock 3 / Farm B	**<0.001**	**<0.001**		0.06	**<0.001**	**<0.001**	**<0.001**	**0.001**	**0.001**	**<0.001**	**<0.001**	**<0.001**
Flock 4 / Farm C	**<0.001**	**<0.001**	0.104		**0.009**	**0.008**	**0.006**	**0.004**	**<0.001**	**0.004**	**0.004**	**0.005**
Flock 5 / Farm B	**<0.001**	**0.001**	0.343	**0.01**		**0.001**	**0.006**	**<0.001**	**<0.001**	**<0.001**	**0.009**	**0.004**
Flock 6 / Farm B	**0.001**	**0.001**	**0.002**	**<0.001**	**0.019**		**<0.001**	**0.001**	**<0.001**	**<0.001**	**0.004**	**0.002**
Flock 7 / Farm C	**<0.001**	**<0.001**	0.18	0.11	0.399	**<0.001**		**0.015**	**<0.001**	**0.003**	**0.011**	**0.014**
Flock 8 / Farm C	**<0.001**	**<0.001**	0.035	**0.013**	0.06	**<0.001**	0.403		**<0.001**	**<0.001**	**0.015**	**0.006**
Flock 9 / Farm D	**<0.001**	**<0.001**	**0.01**	**<0.001**	**0.031**	**0.003**	**0.006**	**<0.001**		**<0.001**	**<0.001**	**0.001**
Flock 10 / Farm D	**<0.001**	**<0.001**	**0.019**	**<0.001**	**0.03**	**<0.001**	**0.028**	**0.001**	0.059		**<0.001**	**<0.001**
Flock 11 / Farm E	**<0.001**	**<0.001**	**0.015**	**<0.001**	**0.042**	**<0.001**	0.112	**0.032**	**<0.001**	**<0.001**		0.09
Flock 12 / Farm E	**<0.001**	**<0.001**	**0.017**	**0.012**	0.077	**<0.001**	0.387	0.412	**0.001**	**<0.001**	**0.019**	

Jaccard

Yue & Clayton

B)	Flock 1 / Farm A	Flock 2 / Farm A	Flock 3 / Farm B	Flock 4 / Farm C	Flock 5 / Farm B	Flock 6 / Farm B	Flock 7 / Farm C	Flock 8 / Farm C	Flock 9 / Farm D	Flock 10 / Farm D	Flock 11 / Farm E	Flock 12 / Farm E
Flock 1 / Farm A		**<0.001**	**<0.001**	**<0.001**	**<0.001**	**<0.001**	**<0.001**	**<0.001**	**<0.001**	**<0.001**	**<0.001**	**<0.001**
Flock 2 / Farm A	**<0.001**		**<0.001**	**<0.001**	**<0.001**	**<0.001**	**<0.001**	**<0.001**	**<0.001**	**<0.001**	**<0.001**	**<0.001**
Flock 3 / Farm B	**<0.001**	**<0.001**		**<0.001**	**<0.001**	**<0.001**	**<0.001**	**<0.001**	**<0.001**	**<0.001**	**<0.001**	**<0.001**
Flock 4 / Farm C	**0.001**	**<0.001**	**<0.001**		**<0.001**	**<0.001**	**<0.001**	**<0.001**	**<0.001**	**<0.001**	**<0.001**	**<0.001**
Flock 5 / Farm B	**<0.001**	**<0.001**	**0.001**	**<0.001**		**<0.001**	**<0.001**	**<0.001**	**<0.001**	**<0.001**	**<0.001**	**<0.001**
Flock 6 / Farm B	**<0.001**	**<0.001**	**<0.001**	**<0.001**	**<0.001**		**<0.001**	**<0.001**	**<0.001**	**<0.001**	**<0.001**	**<0.001**
Flock 7 / Farm C	**<0.001**	**<0.001**	**<0.001**	**<0.001**	**0.003**	**<0.001**		**<0.001**	**<0.001**	**<0.001**	**<0.001**	**<0.001**
Flock 8 / Farm C	**<0.001**	**<0.001**	**<0.001**	**<0.001**	**<0.001**	**<0.001**	**<0.001**		**<0.001**	**<0.001**	**<0.001**	**<0.001**
Flock 9 / Farm D	**<0.001**	**<0.001**	**0.001**	**<0.001**	**<0.001**	**<0.001**	**<0.001**	**<0.001**		**<0.001**	**<0.001**	**<0.001**
Flock 10 / Farm D	**<0.001**	**<0.001**	**<0.001**	**<0.001**	**<0.001**	**0.001**	**<0.001**	**<0.001**	**<0.001**		**<0.001**	**<0.001**
Flock 11 / Farm E	**<0.001**	**<0.001**	**<0.001**	**<0.001**	**<0.001**	0.059	**<0.001**	**<0.001**	**<0.001**	**<0.001**		**<0.001**
Flock 12 / Farm E	**<0.001**	**<0.001**	**<0.001**	**<0.001**	**0.007**	**0.001**	**<0.001**	**0.004**	**<0.001**	**<0.001**	**0.002**	

Jaccard

Yue & Clayton

P value under significant level (p < 0.05) are represented in bold
